# Predictive value of tumor mutational burden for immunotherapy in non-small cell lung cancer: A systematic review and meta-analysis

**DOI:** 10.1371/journal.pone.0263629

**Published:** 2022-02-03

**Authors:** Guangxian Meng, Xiaowei Liu, Tian Ma, Desheng Lv, Ge Sun

**Affiliations:** Department of Thoracic surgery, The Second Hospital of Dalian Medical University, Dalian, Liaoning Province, China; Hamad Medical Corporation, QATAR

## Abstract

**Background:**

Immunotherapy has emerged as a promising treatment for non-small cell lung cancer (NSCLC). Yet, some patients cannot benefit from immunotherapy, and reliable biomarkers for selecting sensitive patients are needed. Herein, we performed a meta-analysis to evaluate the predictive value of tumor mutational burden (TMB) in NSCLC patients treated with immunotherapy.

**Methods:**

Eligible studies were comprehensively searched from electronic databases prior to August 31, 2021. Meta-analyses of high TMB versus low TMB as well as immunotherapy versus chemotherapy in patients with high/low TMB were conducted. Hazard ratio (HR) with corresponding 95% confidence interval (95%CI) for progression-free survival (PFS) and overall survival (OS) and odds ratio (OR) with 95%CI for objective response rate (ORR) were calculated.

**Results:**

A total of 31 datasets (3437 patients) and 6 randomized controlled trials (3662 patients) were available for meta-analyses of high TMB versus low TMB and immunotherapy versus chemotherapy, respectively. High TMB predicted significantly favorable PFS (HR = 0.54, 95%CI: 0.46–0.63, P<0.001) and OS (HR = 0.70, 95%CI: 0.57–0.87, P = 0.001), and higher ORR (OR = 3.14, 95%CI: 2.28–4.34, P<0.001) compared with low TMB. In patients with high TMB, immunotherapy was associated with improved PFS (HR = 0.62, 95%CI: 0.53–0.72), OS (HR = 0.67, 95%CI: 0.57–0.79) and ORR (OR = 2.35, 95%CI: 1.74–3.18) when compared with chemotherapy. However, in patients with low TMB, immunotherapy seemed to predict inferior PFS (HR = 1.20, 95%CI: 1.02–1.41) and ORR (OR = 0.61, 95%CI: 0.44–0.84) and have no OS benefit (HR = 0.88, 95%CI: 0.74–1.05) as compared with chemotherapy.

**Conclusion:**

This meta-analysis demonstrates more clinical benefits concerning treatment response and survival outcomes in high-TMB NSCLC patients who are treated with immunotherapy. TMB is a promising biomarker for discriminating NSCLC patients who can benefit more from immunotherapy.

## Introduction

Lung cancer is one of the most prevalent malignant tumor diseases worldwide and causes tremendous loss of lives accounting for 18.4% of cancer-related death [1]. Most of the newly-diagnosed lung cancers are non-small cell lung cancer (NSCLC). Traditional treatments of NSCLC include surgery, radiotherapy and chemotherapy, but the prognosis of advanced disease is still poor. In recent decades, molecular targeted therapy has greatly improved the treatment response and survivals of advanced NSCLC and is recommended as first-line therapy for patients with activating mutations of cancer-driven genes [2,3]. However, most patients will finally develop drug resistance to targeted therapy [4,5].

Recently, immunotherapy has emerged as an innovative therapy of diverse cancer types with great efficacy. The most representative immunotherapy is immune checkpoint inhibitors (ICIs), which mainly include blockades for programmed cell death 1 (PD-1), programmed cell death ligand 1 (PD-L1) and cytotoxic T-lymphocyte associated protein 4 (CTLA-4). These inhibitors, alone or in combination, improve treatment response and prolong the survival time of NSCLC patients, which show superior efficacy to chemotherapy [6–8]. Moreover, the addition of ICIs to chemotherapy improved survival outcomes compared with chemotherapy alone [9]. The selection of patients with the best clinical benefits from immunotherapy is vital, but the problem is still not well resolved. Till today, PD-L1 expression on tumor tissue has been the only officially approved biomarker for patient selection. However, a subset of patients with low or negative PD-L1 expression could also respond to ICIs [10]. Besides, there is substantial heterogeneity in the spatial and temporal patterns of PD-L1 expression, suggesting that PD-L1 alone is not sufficient enough for patient selection [11]. Apart from ICIs, the other immunotherapy methods, including adoptive cell transfer, antigen-specific cancer vaccines and active immunotherapy, have shown limited success in NSCLC patients [12,13]. Thus, new biomarkers are still needed for screening patients suitable for immunotherapy.

Several candidate biomarkers have been recognized, including PD-L1 expression, cancer-driven mutations, tumor-infiltrating lymphocytes (TILs) and microsatellite instability (MSI), of which PD-L1 expression is officially approved while the standardization for TILs is difficult to be established [14,15]. Tumor mutational burden (TMB), defined as the total number of somatic mutations per Megabase, is emerged as a promising biomarker for patient stratification. Rizvi *et al* firstly determined the mutational landscape of NSCLC patients treated with PD-1 blockades, and found that high-TMB patients had significantly improved response rate and progression-free survival (PFS) than low-TMB patients [16]. The predictive value of high TMB was furtherly validated by the other cohorts [17–19]. Furthermore, several randomized clinical trials (RCTs), including CheckMate-227 [20], IMpower110 [21], POPLAR and OAK [22], showed significant differences in response and survivals favoring immunotherapy over chemotherapy in high-TMB patients but no difference in low-TMB patients.

However, some studies yielded inconsistent results, leading to controversies on the clinical significance of TMB in predicting immunotherapy efficacy. The B-F1RST trial, by recruiting advanced NSCLC patients who were treated with first-line atezolizumab, showed no significant survival difference between high and low blood-based TMB groups [23]. In CheckMate-026 [24], there was no difference in overall survival between nivolumab and chemotherapy in both high-TMB and low-TMB groups. Surprisingly, in the low-TMB group of CheckMate-026 and MYSTIC trials [24,25], patients who were assigned to immunotherapy had shorter PFS and lower response rates than those who were assigned to platinum-based chemotherapy. The discordance may be caused by differences in sample size, study design, measuring assay and algorithms of TMB, threshold value, and inhibitor types. Here, we performed a meta-analysis, by incorporating existing clinical evidence, to evaluate the predictive value of TMB in NSCLC patients treated with immunotherapy.

## Methods

### Selection of eligible studies

This meta-analysis was performed in accordance with the Preferred Reporting Items for Systematic Reviews and Meta-Analysis (PRISMA) statement. Literature databases, including PubMed, EMBASE, Web of Science, Cochrane Library, and Clinicaltrails.gov were searched from inception to August 31, 2021. We used the following free words and their combinations for literature search: (PD-1 OR PD-L1 OR CTLA-4 OR ipilimumab OR tremelimumab OR nivolumab OR pembrolizumab OR lambrolizumab OR atezolizumab OR avelumab OR durvalumab OR “immune checkpoint inhibitor” OR “immune checkpoint inhibitors” OR “ICI” OR “ICIs” OR “immune checkpoint blocker” OR “immune checkpoint blockers” OR “ICB” OR “ICBs”) AND (mutation burden OR mutational burden OR mutation load OR mutational load OR TMB OR TML) AND (lung cancer OR non-small cell lung cancer OR lung adenocarcinoma OR NSCLC). References of candidate articles were manually searched to identify additional eligible studies that were potentially missed from the literature search.

Eligible studies should meet the following criteria: (1) for meta-analysis of high TMB versus low TMB, patients were diagnosed with NSCCL and treated with ICIs, i.e. inhibitors of PD-1/PD-L1, CTLA-4 and their combinations; (2) for meta-analysis of ICIs versus chemotherapy in high/low TMB groups, studies were randomized controlled trials (RCTs) with patients in each arm treated with ICIs or any kind of chemotherapy; (3) TMB was well-defined and measured by whole-exome sequencing (WES) or targeted next-generation sequencing (NGS) panels; (4) studies compared progression-free survival (PFS)/ overall survival (OS)/ objective response rate (ORR) between high TMB and low TMB groups, or between ICIs and chemotherapy in high/low TMB groups; (5) studies reported hazard ratio (HR) and corresponding 95% confidence interval (95%CI) for PFS and OS, or provided original survival data to calculate and survival curves to estimate HR and 95%CI, or provided sufficient data to calculate odds ratio (OR) and 95%CI for ORR. If patients who were treated with ICIs received concurrent non-ICIs, the study should be discarded. For overlapping studies, only the one with the most complete information was selected. Reviews, case reports, meeting abstracts and comments were excluded.

### Data extraction and quality assessment

The following information of each eligible study was extracted: first author, publication year, country of the study, data source, sample size, immunotherapy drug, therapy line, sample source, TMB detection method, TMB definition and cutoff, number of patients in high and low TMB groups, number of patients in ICIs arm and chemotherapy arm, HR and 95%CI for PFS and OS, ORR in each TMB group and arm.

The quality of studies comparing high TMB versus low TMB was assessed by Newcastle-Ottawa Scale (NOS). A total of 9 stars are awarded to items with regard to selection, comparability and outcome category. Studies with 7 or more stars are considered of high quality. Besides, the quality of RCTs comparing ICIs with chemotherapy was assessed by Cochrane Collaboration’s tool for assessing risk of bias. The selection bias (random sequence generation, allocation concealment), performance bias (blinding of participants and personnel), detection bias (blinding of outcome assessment), attrition bias (incomplete outcome data) and reporting bias (selective reporting) were graded as of low, high or unclear risk.

Two researchers performed the literature search, study selection, data extraction and quality assessment independently. Discrepancies, if occurred, were solved by further discussion.

### Statistical analysis

Between-study heterogeneity was assessed by I^2^ statistics and graded as low (<25%), medium (25~50%) and high (>50%) level. The present meta-analysis was divided into two parts: the meta-analysis of high TMB versus low TMB, and the meta-analysis of immunotherapy versus chemotherapy in high/low TMB groups. The first part was to explore the predictive value of TMB in NSCLC patients treated with immunotherapy. The second part was to investigate whether TMB level would modify the efficacy of immunotherapy versus chemotherapy in NSCLC patients. The effect sizes, including HR for survival outcomes and OR for treatment response, and their corresponding 95%CI were pooled together. However, to obtain more conservative results, all effect sizes were pooled by using the random-effect model regardless of between-study heterogeneity. Subgroup analysis was performed according to the region (Asian, Western), data source (clinical trial, cohort), ICIs category (PD-1/PD-L1 inhibitors, PD-(L)1 inhibitors plus CTLA-4 inhibitors), treatment line (1, others), sample source (tumor, blood), TMB detection method (WES, targeted NGS), sample size (≥100, <100), TMB cutoff (≥16 or <16 mut/Mb). Meta-regression analysis regarding sample size and sensitivity analysis were also conducted. Publication bias was assessed by both viewing the symmetry of funnel plots and Egger’s test. All the analyses were performed by using STATA 14.0 (Stata Corporation, TX, USA). P<0.05 indicated statistical significance.

## Results

### Study characteristics

**Fig 1:**
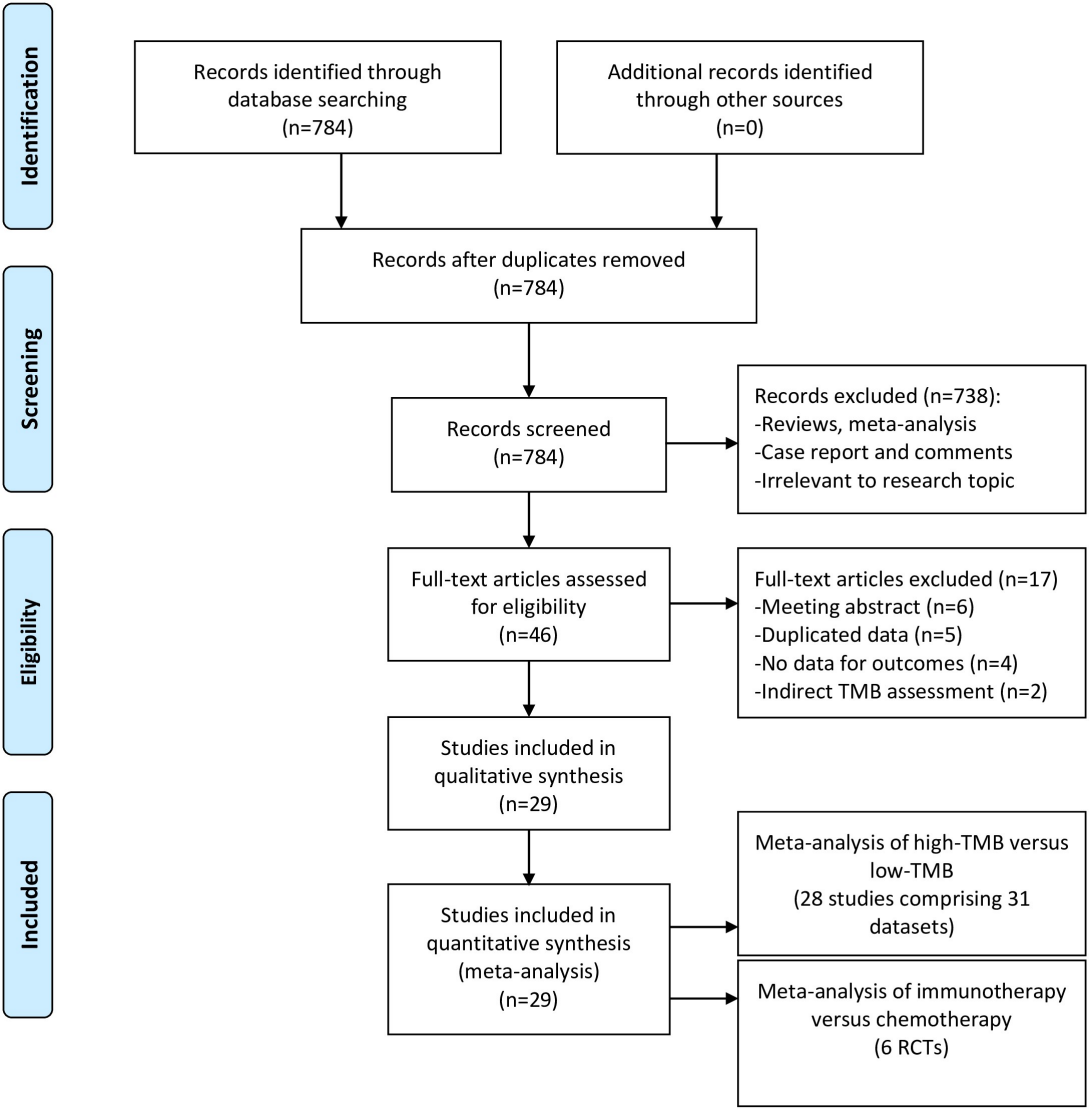
Flowchart of literature search.

We finally identified 28 eligible studies (31 datasets, 3437 NSCLC patients evaluable for TMB) for the meta-analysis of high TMB versus low TMB [16–20,22–44] as shown in Fig 1. There were 1140 patients in the high-TMB group and 2297 in the low-TMB group. Most of the datasets were from cohort studies and 10 were from clinical trials. Monotherapy of PD-(L)1 inhibitors, combination therapy of PD-(L)1 plus CTLA-4 inhibitors and various ICIs were administered in 24, 4 and 3 datasets, respectively. Tumor or blood samples were selected for TMB detection in 21 and 10 datasets, respectively. Only 8 datasets applied WES and the others used commercial or in-house targeted NGS panels for TMB detection. PFS, OS and ORR were assessed in 27, 19 and 19 datasets, respectively. All studies were considered of high quality according to NOS (S1 Table). The baseline characteristics of these datasets were summarized in Table 1.

**Table 1:** Baseline characteristics of studies in meta-analysis comparing high TMB group with low TMB group in NSCLC patients receiving immunotherapy.

Author	Year	Country	Data source	Immunotherapy	Therapy line	Sample source	TMB detection method	TMB cutoff	Sample size^&^ (High/low TMB)	Outcome
Rizvi N	2015	USA	Cohort	Pembrolizumab	≥1	Tumor	WES	178 mutations	34 (17/17)	PFS, ORR
Carbone D	2017	Various	Clinical trial	Nivolumab	1	Tumor	WES	243 mutations	158 (47/111)	PFS, OS, ORR
Goodman A	2017	USA	Cohort	PD-1/PD-L1 inhibitors	≥2	Tumor	FoundationOne	20 mut/Mb	36 (3/33)	PFS, OS, ORR
Rizvi H	2018	USA	Cohort	Various ICIs	≥1	Tumor	MSK-IMPACT	7.4 mut/Mb, median	240 (119/121)	PFS
Hellmann M, CheckMate-012	2018	Various	Clinical trial	Nivolumab plus ipilimumab	1	Tumor	WES	158 mutations, median	75 (37/38)	PFS, ORR
Hellmann M, CheckMate-227	2018	Various	Clinical trial	Nivolumab plus ipilimumab	1	Tumor	FoundationOne	10 mut/Mb	330 (139/191)	PFS
Gandara D, OAK trial	2018	Various	Clinical trial	Atezolizumab	≥2	Blood	FoundationOne	16 mut/Mb	293 (77/216)	PFS, OS, ORR
Gandara D, POPLAR trial	2018	Various	Clinical trial	Atezolizumab	≥2	Blood	FoundationOne	16 mut/Mb	105 (25/80)	PFS, OS, ORR
Chae Y	2019	USA	Cohort	PD-1/PD-L1 inhibitors	≥1	Tumor	FoundationOne	15 mut/Mb	34 (11/23)	PFS, OS
Samstein R	2019	USA	Cohort	Various ICIs	NA	Tumor	MSK-IMPACT	Top 20%	350 (70/280)	OS
Ready N	2019	Various	Clinical trial	Nivolumab plus ipilimumab	1	Tumor	FoundationOne	10 mut/Mb	98 (48/50)	PFS, ORR
Wang Z	2019	China	Cohort	PD-1/PD-L1 inhibitors	≥1	Blood	NCC-GP150	6 mut/Mb	50 (28/22)	PFS, ORR
Fang W	2019	China	Clinical trial	PD-1/PD-L1 inhibitors	≥2	Tumor	WES	157 mutations, top33%	73 (25/48)	PFS, ORR
Ohue Y	2019	Japan	Cohort	Nivolumab, pembrolizumab	≥1	Tumor	WES	178 mutations, median	11 (4/7)	PFS, OS, ORR
Heeke S	2019	France	Cohort	Nivolumab, pembrolizumab	1, 2	Tumor	FoundationOne	15 mut/Mb	36 (15/21)	PFS
Alborelli I	2020	Switzerland	Cohort	PD-1/PD-L1 inhibitors	≥1	Tumor	Oncomine TML assay	9 mut/Mb	76 (25/51)	PFS, OS, ORR
Wang Z	2020	China	Cohort	PD-1/PD-L1 inhibitors	≥1	Blood	NCC-GP150	6 mut/Mb	64 (28/36)	OS
Hurkmans D	2020	Netherland	Cohort	Nivolumab	≥2	Tumor	Oncomine TML assay	11 mut/Mb	25 (8/17)	PFS, OS
Huang D	2020	China	Cohort	PD-1/PD-L1 inhibitors	≥1	Tumor	GeneseeqOne	10 mut/Mb	14 (7/7)	PFS, OS, ORR
Aggarwal C	2020	USA	Cohort	Pembrolizumab	1	Blood	GuardantOMNI	16 mut/Mb	26 (14/12)	PFS, OS
Rizvi N, D mono^#^	2020	Various	Clinical trial	Durvalumab	1	Blood	GuardantOMNI	20 mut/Mb	286 (77/209)	PFS, OS, ORR
Rizvi N, D+T^#^	2020	Various	Clinical trial	Durvalumab plus tremelimumab	1	Blood	GuardantOMNI	20 mut/Mb	268 (64/204)	PFS, OS, ORR
Shim J, cohort 1	2020	South Korea	Cohort	PD-1/PD-L1 inhibitors	≥1	Tumor	WES	272 mutations, top 25%	198 (47/151)	PFS, OS, ORR
Shim J, cohort 2	2020	USA	Cohort	Various ICIs	NA	Tumor	WES	272 mutations, top 25%	89 (30/59)	PFS, OS
Xu Y	2020	China	Cohort	PD-L1 inhibitors	≥1	Tumor	Targeted NGS	10.62 mut/Mb, mean	53 (25/28)	PFS, OS
B-F1RST study	2020	Various	Clinical trial	PD-L1 inhibitors	1	Blood	Targeted NGS	16 mut/Mb	119 (28/91)	PFS, OS, ORR
Chen X	2021	China	Cohort	PD-1/PD-L1 inhibitors	≥1	Blood	OncoScreen	7 mut/Mb	42 (12/30)	PFS
Ma Y	2021	China	Cohort	PD-1 inhibitors	2	Blood	Targeted NGS	6 mut/Mb	13 (6/7)	PFS, ORR
Pabla S	2021	USA	Cohort	PD-1/PD-L1 inhibitors	NA	Tumor	Targeted NGS	10 mut/Mb	110 (56/54)	OS, ORR
Kim H	2021	South Korea	Cohort	PD-1/PD-L1 inhibitors	≥1	Tumor	Targeted NGS	5.29 mut/Mb, median	30 (15/15)	PFS
Yoh K	2021	Japan	Cohort	PD-1/PD-L1 inhibitors	≥1	Tumor	WES	200 mutations	101 (33/68)	ORR

^&^ Number of patients evaluable for TMB.

^#^ D mono indicates durvalumab monotherapy and D+T indicates durvalumab plus tremelimumab combination therapy.

NSCLC: Non-small cell lung cancer; ICIs: Immune checkpoint inhibitors; PD-1: Programmed cell death 1; PD-L1: Programmed cell death-ligand 1; TMB: Tumor mutation burden; WES: Whole-exome sequencing; NGS: Next-generation sequencing; PFS: Progression-free survival; OS: Overall survival; ORR: Objective response rate; mut/Mb: Numbers of mutation per Megabase; NA: Not available.

In addition, we identified 6 RCTs comprising 3663 patients, including CheckMate-026 [24], CheckMate-227 [6,20], MYSTIC [25], Impower110 [21], POPLAR and OAK [22], for the meta-analysis of immunotherapy versus chemotherapy. In the first four trials, patients in the immunotherapy group were treated with first-line PD-(L)1 inhibitors alone or combined with CTLA-4 inhibitor, and patients in another group received platinum-based chemotherapy. Whereas, the last two trials recruited previously treated patients and randomized patients to atezolizumab and docetaxel arms. The details of these clinical trials were shown in Table 2. According to Cochrane Collaboration’s tool for assessing risk of bias, all trials had a low risk of bias in most domains (S2 Table).

**Table 2:** Baseline characteristics of studies in meta-analysis comparing immunotherapy with chemotherapy in NSCLC patients with high or low TMB.

Trial	Treatment		Treatment line	Sample source	TMB detection method	TMB cutoff	Sample size (arm 1/arm 2)		Outcome
	Arm 1	Arm 2					High TMB	Low TMB	
CheckMate-026	Nivolumab	CT	1	Tumor	WES	243 mutations	107 (47/60)	205 (111/94)	PFS, OS, ORR
CheckMate-227	Nivolumab plus ipilimumab	CT	1	Tumor	FoundationOne	10 mut/Mb	299 (139/160)	380 (191/189)	PFS, OS, ORR
POPLAR	Atezolizumab	Docetaxel	>1	Blood	FoundationOne	16 mut/Mb	63 (25/38)	148 (80/68)	PFS, OS, ORR
OAK	Atezolizumab	Docetaxel	>1	Blood	FoundationOne	16 mut/Mb	158 (77/81)	425 (216/209)	PFS, OS, ORR
MYSTIC, D vs. CT	Durvalumab	CT	1	Blood	GuardantOMNI	20 mut/Mb	147 (77/70)	394 (209/185)	PFS, OS, ORR
MYSTIC, D+T vs. CT	Durvalumab plus tremelimumab	CT	1	Blood	GuardantOMNI	20 mut/Mb	134 (64/70)	389 (204/185)	PFS, OS, ORR
IMpower110	Atezolizumab	CT	1	Blood	FoundationOne	16 mut/Mb	87	302	PFS, OS

NSCLC: Non-small cell lung cancer; CT: Chemotherapy; TMB: Tumor mutation burden; WES: Whole-exome sequencing; PFS: Progression-free survival; OS: Overall survival; ORR: Objective response rate; mut/Mb: Mutations per megabase.

### High TMB versus low TMB in patients treated with immunotherapy

**Fig 2:**
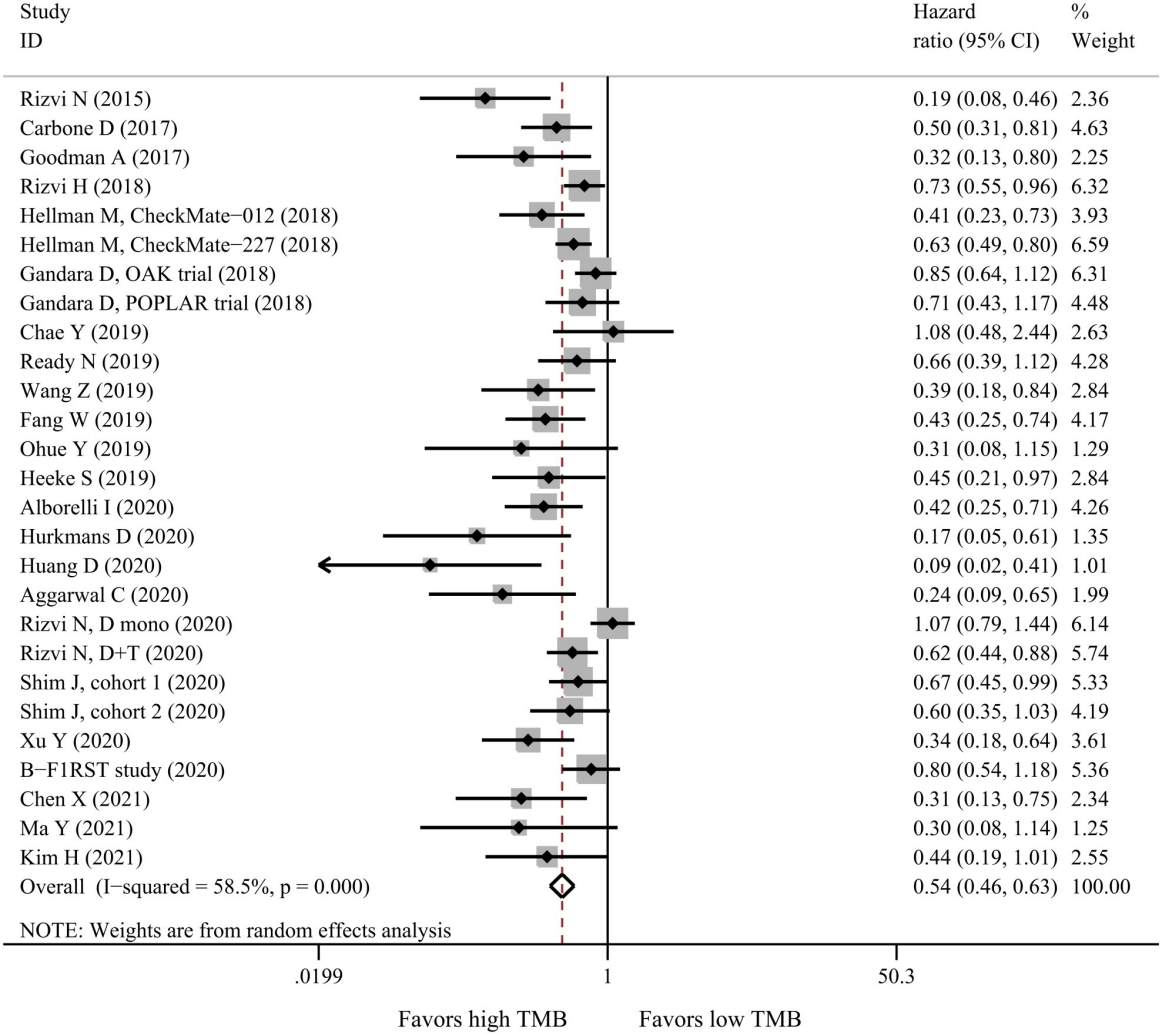
Forest plot for PFS of high-TMB versus low-TMB in NSCLC patients receiving immunotherapy. PFS: Progression-free survival; TMB: Tumor mutational burden; NSCLC: Non-small cell lung cancer.

The predictive value of TMB in patients receiving immunotherapy was investigated by pooling together the survival and response outcomes of all eligible studies (S3 Table). Meta-analysis of 27 datasets comprising 2812 patients demonstrated that patients with high TMB had favorable PFS (HR = 0.54, 95%CI: 0.46–0.63, P<0.001, I^2^ = 58.5%, Fig 2) compared with those with low TMB. Stratified analyses according to baseline features showed that high TMB predicted prolonged PFS in all subgroups except for PD-L1 inhibitors (Table 3). High TMB was associated with longer PFS in patients treated with only PD-L1 inhibitors (HR = 0.76, 95%CI: 0.57–1.02, P = 0.070), but the association did not reach statistical significance which may be due to substantial between-study heterogeneity.

**Table 3:** Subgroup analysis of immunotherapy efficacy for NSCLC in high TMB group versus low TMB group.

Subgroup	PFS				OS				ORR			
	No	I^2^ (%)	HR (95%CI)	P	No	I^2^ (%)	HR (95%CI)	P	No	I^2^ (%)	OR (95%CI)	P
Region												
Asian	9 (484)	24.1	0.41 (0.31–0.55)	<0.001	5 (340)	64.6	0.59 (0.32–1.11)	0.100	7 (460)	0	3.21 (2.01–5.11)	<0.001
Western	18 (2328)	59.8	0.6 (0.50–0.72)	<0.001	14 (1975)	56.9	0.72 (0.57–0.91)	0.005	12 (1658)	58.4	3.10 (2.04–4.73)	<0.001
Data source												
Clinical trial	10 (1805)	53.8	0.68 (0.56–0.81)	<0.001	6 (1229)	6.2	0.90 (0.76–1.06)	0.220	9 (1475)	49.5	3.29 (2.20–4.92)	<0.001
Cohort	17 (1007)	52.3	0.42 (0.33–0.54)	<0.001	13 (1086)	56.3	0.55 (0.39–0.78)	0.001	10 (643)	41.3	3.04 (1.70–5.42)	<0.001
ICIs category												
PD-1/PD-L1 inhibitors	21 (1712)	66.3	0.48 (0.38–0.61)	<0.001	16 (1608)	53.3	0.75 (0.59–0.95)	0.018	16 (1677)	37.5	2.71 (1.91–3.85)	<0.001
PD-(L)1 inhibitors plus CTLA-4 inhibitors	4 (771)	0	0.61 (0.51–0.72)	<0.001	1 (268)	-	0.68 (0.47–0.99)	0.046	3 (441)	0	5.25 (3.26–8.47)	<0.001
Various ICIs	2 (329)	0	0.70 (0.55–0.90)	0.005	2 (439)	0	0.49 (0.35–0.69)	<0.001	0	-	-	-
PD-1/PD-L1 inhibitors category												
PD-1 inhibitors	7 (303)	3.4	0.35 (0.25–0.49)	<0.001	4 (220)	40.8	0.57 (0.31–1.05)	0.072	4 (216)	23.3	5.18 (2.02–13.27)	0.001
PD-L1 inhibitors	5 (856)	63.6	0.76 (0.57–1.02)	0.070	5 (856)	63.7	0.82 (0.59–1.15)	0.249	4 (803)	60.3	2.62 (1.34–5.11)	0.005
Treatment line												
1	8 (1360)	62.8	0.64 (0.50–0.81)	<0.001	5 (857)	0.5	0.81 (0.67–0.99)	0.037	6 (1004)	59.8	4.01 (2.36–6.82)	<0.001
Others	19 (1452)	56.4	0.48 (0.39–0.60)	<0.001	14 (1458)	64.9	0.64 (0.47–0.88)	0.005	13 (1114)	27.4	2.59 (1.74–3.85)	<0.001
Sample source												
Tumor	18 (1510)	45.9	0.50 (0.41–0.60)	<0.001	12 (1154)	61.8	0.56 (0.39–0.80)	0.002	12 (984)	33.1	3.00 (2.00–4.49)	<0.001
Blood	9 (1202)	61.6	0.65 (0.50–0.85)	0.002	7 (1161)	7.9	0.88 (0.74–1.05)	0.152	7 (1134)	60.2	3.48 (1.96–6.20)	<0.001
TMB detection method												
WES	7 (638)	28.6	0.48 (0.37–0.62)	<0.001	4 (456)	42.3	0.81 (0.53–1.24)	0.336	7 (650)	0	3.27 (2.24–4.79)	<0.001
Targeted NGS	20 (2174)	60.9	0.57 (0.47–0.69)	<0.001	15 (1859)	60.5	0.67 (0.53–0.87)	0.002	12 (1468)	56.9	3.10 (1.94–4.95)	<0.001
Sample size												
≥100	9 (1997)	35.4	0.73 (0.64–0.84)	<0.001	9 (1887)	55.1	0.88 (0.71–1.09)	0.234	9 (1638)	59.4	2.51 (1.66–3.79)	<0.001
<100	18 (815)	21.4	0.41 (0.33–0.50)	<0.001	10 (428)	0	0.46 (0.35–0.62)	<0.001	10 (480)	0	4.83 (3.04–7.67)	<0.001
TMB cutoff of NGS												
≥16 mut/Mb	7 (1133)	61.2	0.71 (0.55–0.93)	0.011	7 (1133)	26.6	0.85 (0.69–1.05)	0.130	6 (1107)	57.5	2.96 (1.70–5.17)	<0.001
<16 mut/Mb	13 (1041)	49.5	0.49 (0.38–0.62)	<0.001	7 (376)	65.8	0.51 (0.29–0.91)	0.023	6 (361)	6.7	3.41 (1.38–8.41)	0.008

No: Number of included studies and patients. NSCLC: Non-small cell lung cancer; ICIs: Immune checkpoint inhibitors; PD-1: Programmed cell death 1; PD-L1: Programmed cell death-ligand 1; CTLA-4: Cytotoxic T lymphocyte associated antigen 4; TMB: Tumor mutation burden; WES: Whole-exome sequencing; NGS: Next-generation sequencing; PFS: Progression-free survival; OS: Overall survival; ORR: Objective response rate; mut/Mb: Numbers of mutation per Megabase; HR: Hazard ratio; OR: Odds ratio; 95%CI: 95% confidence interval.

**Fig 3:**
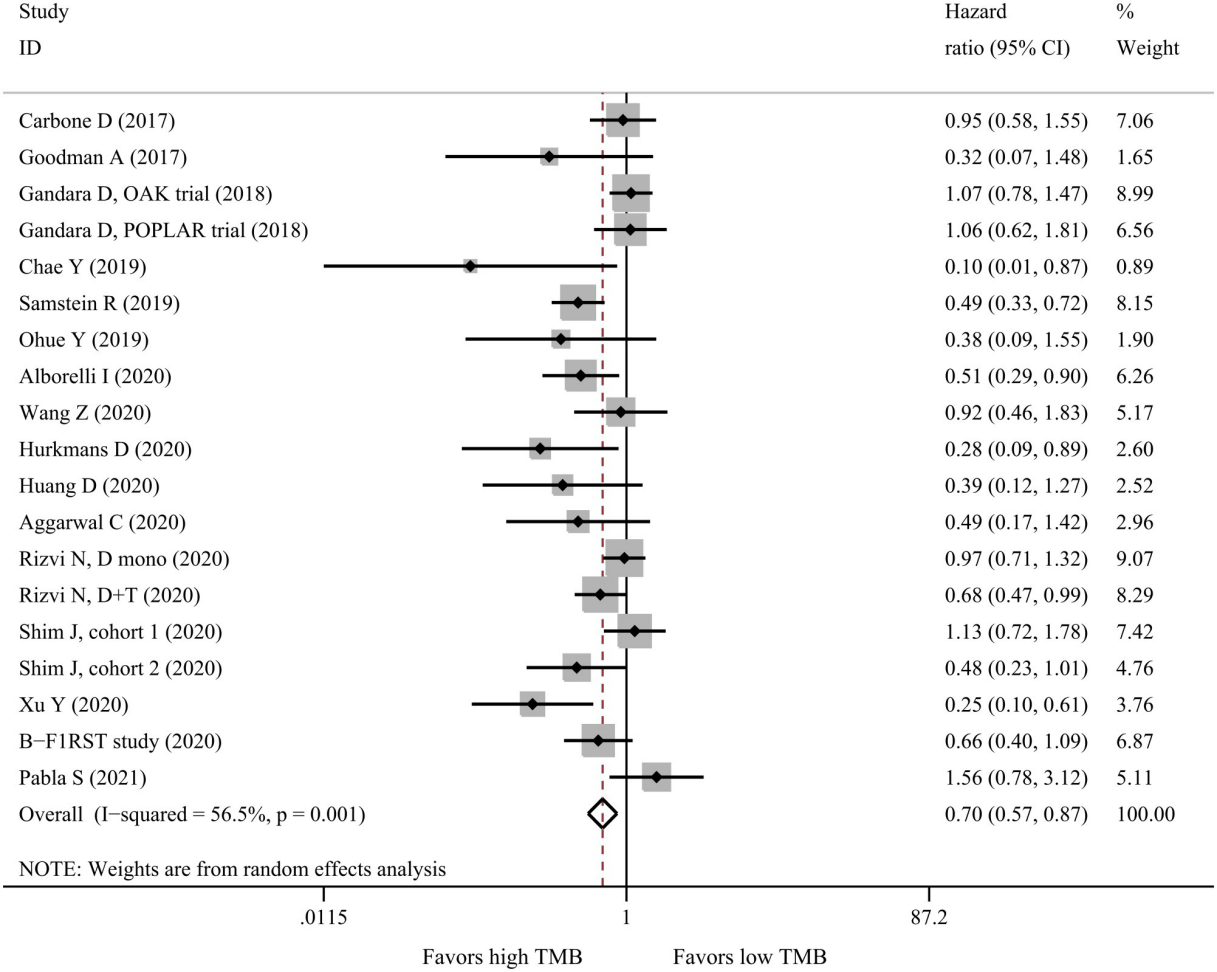
Forest plot for OS of high-TMB versus low-TMB in NSCLC patients receiving immunotherapy. OS: Overall survival; TMB: Tumor mutational burden; NSCLC: Non-small cell lung cancer.

Meta-analysis of 19 datasets comprising 2315 patients showed that high TMB predicted significantly better OS than low TMB (HR = 0.70, 95%CI: 0.57–0.87, P = 0.001, I^2^ = 56.5%, Fig 3). However, further analyses yielded inconsistent results in subgroups of clinical trials, PD-L1 inhibitors, blood samples, sample size ≥100 and TMB cutoff ≥16 mut/Mb, in which high TMB was not significantly associated with OS (Table 3).

**Fig 4:**
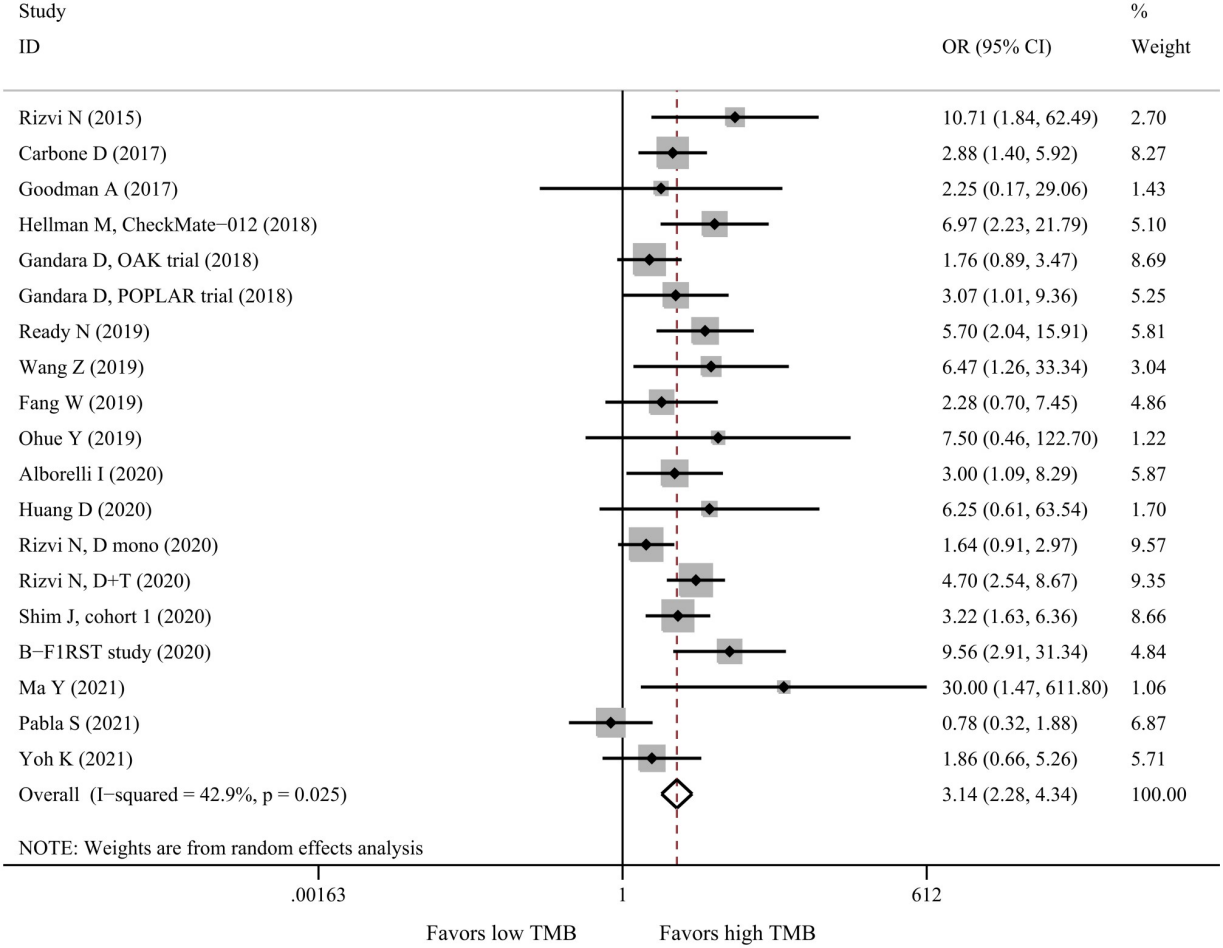
Forest plot for ORR of high-TMB versus low-TMB in NSCLC patients receiving immunotherapy. OR: Objective response rate; TMB: Tumor mutational burden; NSCLC: Non-small cell lung cancer; OR: Odds ratio.

By pooling 19 datasets with 2118 patients, we found that the response rate was significantly higher in the high-TMB group than in the low-TMB group (OR = 3.14, 95%CI: 2.28–4.34, P<0.001, I^2^ = 42.9%, Fig 4). Furthermore, all subgroup analyses showed consistent results with the overall analysis (Table 3).

### Immunotherapy versus chemotherapy in high-TMB/low-TMB groups

**Fig 5:**
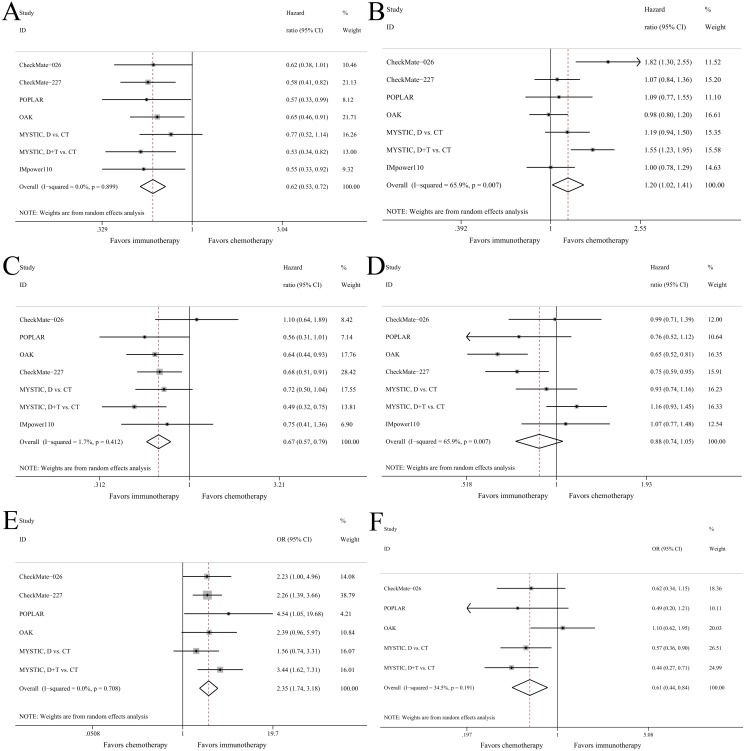
Forest plots of meta-analysis of immunotherapy versus chemotherapy in NSCLC patients with high or low TMB. (A) PFS in high-TMB patients; (B) PFS in low-TMB patients; (C) OS in high-TMB patients; (D) OS in low-TMB patients; (E) ORR in high-TMB patients; (F) ORR in low-TMB patients.

The survival and response outcomes in all RCTs comparing immunotherapy versus chemotherapy in NSCLC patients with high or low TMB were summarized (S4 Table). In the high-TMB group with 1114 patients, there was almost no between-study heterogeneity. Compared with chemotherapy, immunotherapy predicted significant better PFS (HR = 0.62, 95%CI: 0.53–0.72, P<0.001, Fig 5A), OS (HR = 0.67, 95%CI: 0.57–0.79, P<0.001, Fig 5C) and higher response rate (OR = 2.35, 95%CI: 1.74–3.18, P<0.001, Fig 5E). The associations remained significant in all subgroups except for tumor sample in OS outcome (S5 Table).

On the contrary, in the low-TMB group with 2623 patients, immunotherapy seemed to predict inferior PFS (HR = 1.20, 95%CI: 1.02–1.41, P = 0.032, Fig 5B) and lower response rate (OR = 0.61, 95%CI: 0.44–0.84, P = 0.002, Fig 5F) than chemotherapy. The OS did not differ between both treatments (HR = 0.88, 95%CI: 0.74–1.05, P = 0.154, Fig 5D). The subgroup of first-line treatment favored chemotherapy over immunotherapy in terms of PFS (HR = 1.27, 95%CI: 1.04–1.56) and ORR (OR = 0.53, 95%CI: 0.39–0.71) as shown in S5 Table.

### Sensitivity analysis and meta-regression analysis

Sensitivity analysis showed that the pooled effect sizes were not affected by any single study in the meta-analysis of high TMB versus low TMB and analysis of immunotherapy versus chemotherapy in high-TMB patients (S1 Fig). However, the results were not robust enough in the analysis of immunotherapy versus chemotherapy in low-TMB patients (S1 Fig). Meta-regression demonstrated that sample size was a potential source of heterogeneity in meta-analysis of high TMB versus low TMB (P for PFS<0.001, P for OS = 0.124, P for ORR = 0.087, S2 Fig).

### Publication bias

The funnel plots for meta-analysis of high TMB versus low TMB were visually asymmetric (S3 Fig), and Egger’s tests suggested obvious publication bias. Whereas, the plots for analysis of immunotherapy versus chemotherapy were symmetric (S4 Fig), and Egger’s tests indicated there was no publication bias.

## Discussion

Neoantigens may contribute to immunogenicity of tumors through eliciting T-cell-dependent immune response [45]. Then, the neoantigen load is considered as a potential biomarker of cancer immunotherapy [46]. Since the accumulation of somatic protein-altering mutations in tumors results in the formation of tumor neoantigens [47], high TMB in tumor tissue may be associated with high neoantigen load and is linked to increased sensitivity to ICIs [48]. Our meta-analysis focused on the predictive value of TMB in NSCLC patients treated with ICIs The analysis of 28 studies comprising 3437 patients demonstrated that high-TMB patients had significantly longer PFS, OS and higher ORR than low-TMB patients. Additional analysis of 6 RCTs revealed that high-TMB patients had superior benefits from immunotherapy to chemotherapy whereas low-TMB patients did not. These results indicate that high TMB is an effective biomarker to select NSCLC patients who are sensitive to immunotherapy.

TMB was previously determined by WES method on tumor tissue (tissue-based TMB, or tTMB for short), which was found to be correlated with PFS in advanced NSCLC patients treated with immunotherapy [16,18,24]. However, the unavailability of sufficient tumor materials is a major limitation for tTMB measurement with only one-third to a half of patients evaluable for tTMB [49]. Recently, commercial kits or inhouse panels targeting cancer-driven genes based on the hybrid NGS method have been gradually applied to detect TMB using blood samples (known as blood-based TMB or bTMB), which is minimally invasive and may represent an alternative method [11]. Qiu *et al* found that two commercial bTMB assays (FoundationOne and GuardantOMNI) were highly correlated and that both NGS-based bTMB assays were correlated with WES-based tTMB in patients with relatively high TMB [50]. Yet, Kuderer *et al*. observed significant discordance between two NGS assays [51]. Thus, the concordances for TMB assessment between WES and NGS methods, between different commercial NGS platforms and between tissue- and blood-based assays are still inconclusive. Subgroup analyses in the present meta-analysis demonstrated that both bTMB and tTMB, as well as WES- and NGS-based TMB, were potential predictors in terms of PFS and ORR. Whereas, we found that bTMB and NGS-based TMB were not significantly associated with OS in NSCLC patients. The concordance between and optimal choice of TMB assessments in terms of sample source (tissue, blood) and platforms (WES, commercial or custom NGS assays) still needs more investigation.

We further explored whether there was an optimal TMB threshold value to discriminate the most sensitive patients for immunotherapy. When divided by 16 mut/Mb, a commonly used threshold of the FDA-approved FoundationOne assay, both subgroups showed prolonged PFS and higher response rates in high-TMB patients than in low-TMB patients. In terms of PFS, the HR magnitude of ≥16 mut/Mb subgroup was significantly larger than that of <16 mut/Mb subgroup (HR = 0.71 versus HR = 0.49). Similar pattern was also observed in the other subgroups of TMB cutoffs that the lower subgroup, at specific cutoff, had smaller HR estimates than the upper subgroup in terms of PFS and OS (S5 Fig). Meta-regression also indicated the HR magnitude for PFS tended to increase with TMB cutoff value (P = 0.086). Surprisingly, there was no significant OS difference in the ≥16 mut/Mb subgroup, which may be caused by the inclusion of clinical trials. Four clinical trials defined high-TMB patients as with ≥16 or 20 mut/Mb [22,23,25] but found no significant difference in terms of PFS and OS between high-TMB and low-TMB patients (S4 Table). Although there is still no consensus on optimal TMB value, large-scale clinical trials rather than cohort studies have suggested that a high TMB threshold may not be able to effectively identify patients sensitive for immunotherapy. Our analysis further suggests that low TMB cutoff may have more discriminating ability for patients suitable for ICIs. Apart from cutoffs, the various targeted NGS kits may have impact on predictive value of TMB (S6 Fig). High TMB by FoundationOne kit was associated with favorable PFS and ORR, and by other kits predicted prolonged survivals and higher ORR. Although no significant benefits were found in high-TMB patients by GuardantOMNI kit, we cannot draw a conclusion due to small number of eligible studies.

Besides of targeted NGS methods that were mostly used, WES method counting the total number of somatic mutations per exome was applied for TMB detection in one-fourth of the included studies. High TMB by WES predicted clinical benefits in terms of PFS and ORR with no significant difference from that by targeted NGS method but had very low between-study heterogeneity. Yet, we found no OS benefit in high-TMB patients defined by WES mothed. Taken together, the present meta-analysis could not decide the preferred choice of WES or targeted NGS for TMB assessment.

Previous meta-analyses found that high TMB was not or only marginally associated with OS in NSCLC [52–55], while the present study observed a significant association between TMB and OS. Nevertheless, the predictive value of TMB for OS needs to be interpreted with caution. The subgroup of clinical trials showed similar OS benefit between high-TMB and low-TMB patients (HR = 0.90, 95%CI 0.76–1.06, P = 0.220), while the cohort subgroup observed superior OS benefit in high-TMB to low TMB patients (HR = 0.55, 95%CI 0.39–0.78, P = 0.005). These large-scale clinical trials, derived from CheckMate-026, B-F1RST, POPLAR and OAK trials, MYSTIC [22–25], all observed no significant association between TMB and OS in NSCLC patients receiving monotherapy of ICIs. Considering the discrepancy between clinical trials and cohort studies, whether high-TMB can predict improved clinical outcomes in NSCLC patients receiving immunotherapy should be clarified in the future by prospective trials designed for this purpose.

In addition to the analysis of high-TMB versus low-TMB, we also compared the clinical efficacy of immunotherapy with chemotherapy in different TMB groups. High-TMB patients had more clinical benefits from immunotherapy than from chemotherapy in terms of PFS, OS and ORR, regardless of ICIs combination (single agents or combination), treatment line (first-line or later) and sample source (tumor tissue or blood). In low-TMB patients, there was no superior benefit for immunotherapy to chemotherapy. Moreover, in the first-line setting, low-TMB patients who were treated with ICIs had significantly worse PFS and lower response rate than those treated with chemotherapy. These results highlight the clinical value of TMB in the management of personalized medicine.

Previous analyses have found a slightly more benefit but a lower rate of immune-related adverse events from anti-PD-1 inhibitors than from anti-PD-L1 inhibitors [56,57], suggesting a preferred choice of ICIs for NSCLC patients. The present meta-analysis implicated more predictive values for TMB in anti-PD-1 inhibitors than in anti-PD-L1 inhibitors by comparing the magnitude of pooled effect sizes (Table 3). The HR estimates of PFS and OS were obviously smaller (0.35 versus 0.76, 0.57 versus 0.82) and the OR estimates of ORR was larger (5.18 versus 2.62) in anti-PD-1 inhibitors than in anti-PD-L1 inhibitors. However, we did not investigate whether TMB was associated with the occurrence of adverse events in NSCLC patients treated with ICIs due to the lack of related studies.

Our meta-analysis has several limitations. First, there was substantial between-study heterogeneity. Although we applied the random-effect model to all analyses and performed further subgroup analyses, the heterogeneity cannot be resolved. Patient-level data rather than study-level data are warranted. Second, we only pooled HR estimates of the univariate analysis which did not adjust for potential confounders, such as PD-L1 status, smoking status, age and gender, since there was a few multivariate analysis. Third, most of the included studies were retrospectively analyzed and not prospectively designed. Fourth, there was obvious publication bias in the meta-analysis of high TMB versus low TMB, indicating the lack of negative results from studies with a small sample size. Fifth, most of the included studies were from Western and East Asian countries, and mutational landscape that may affect immunotherapy response and immunotherapies in NSCLC were rare reported in the other countries [58]. Collectively, large-scale, prospectively designed clinical trials recruiting participants with diverse genetic background and aiming at the predictive value of TMB for immunotherapy are needed.

In conclusion, the present meta-analysis provides evidence that NSCLC patients with high TMB have more clinical benefits from immunotherapy than those with low TMB and those treated with chemotherapy. TMB is a promising biomarker for NSCLC patients receiving immunotherapy and can be used in clinical practice to identify the most sensitive patients for immunotherapy.

## Supporting information

S1 Checklist (DOCX)

S1 Fig Sensitivity analysis of immunotherapy versus chemotherapy in NSCLC patients with high or low TMB. (A) PFS in high-TMB patients; (B) PFS in low-TMB patients; (C) OS in high-TMB patients; (D) OS in low-TMB patients; (E) ORR in high-TMB patients; (F) ORR in low-TMB patients.
(TIF)

S2 Fig Meta-regression analysis of high-TMB versus low-TMB in immunotherapy-treated NSCLC for associations between sample size and PFS (A), OS (B) and ORR (C). (TIF)

S3 Fig Funnel plots of high-TMB versus low-TMB in immunotherapy-treated NSCLC for PFS (A), OS (B) and ORR (C). (TIF)

S4 Fig Funnel plots of meta-analysis of immunotherapy versus chemotherapy. (A) PFS in high-TMB patients; (B) PFS in low-TMB patients; (C) OS in high-TMB patients; (D) OS in low-TMB patients; (E) ORR in high-TMB patients; (F) ORR in low-TMB patients.
(TIF)

S5 Fig Forest plots of PFS, OS and ORR in subgroup analyses according to various TMB thresholds. (TIFF)

S6 Fig Forest plots of PFS, OS and ORR in subgroup analyses of different targeted NGS kits. (TIFF)

S1 Table NOS assessment of studies in meta-analysis of high TMB versus low TMB in NSCLC patients treated with immunotherapy. (DOCX)

S2 Table Quality assessment of studies in meta-analysis of immunotherapy versus chemotherapy using Cochrane Collaboration’s tool for assessing risk of bias. (DOCX)

S3 Table Survival and response outcomes in meta-analysis of high TMB versus low TMB group in NSCLC patients receiving immunotherapy. (DOCX)

S4 Table Survival and response outcomes in meta-analysis of immunotherapy versus chemotherapy in NSCLC patients with high or low TMB. (DOCX)

S5 Table Subgroup analysis of immunotherapy versus chemotherapy in NSCLC patients with high or low TMB. (DOCX)
