# Supplementary figures and images for: Predictive value of tumor mutational burden for immunotherapy in non-small cell lung cancer: A systematic review and meta-analysis
Source: PLoS One. 2022 Feb 3;17(2):e0263629. doi: 10.1371/journal.pone.0263629 (PMC8812984; doi:10.1371/journal.pone.0263629)

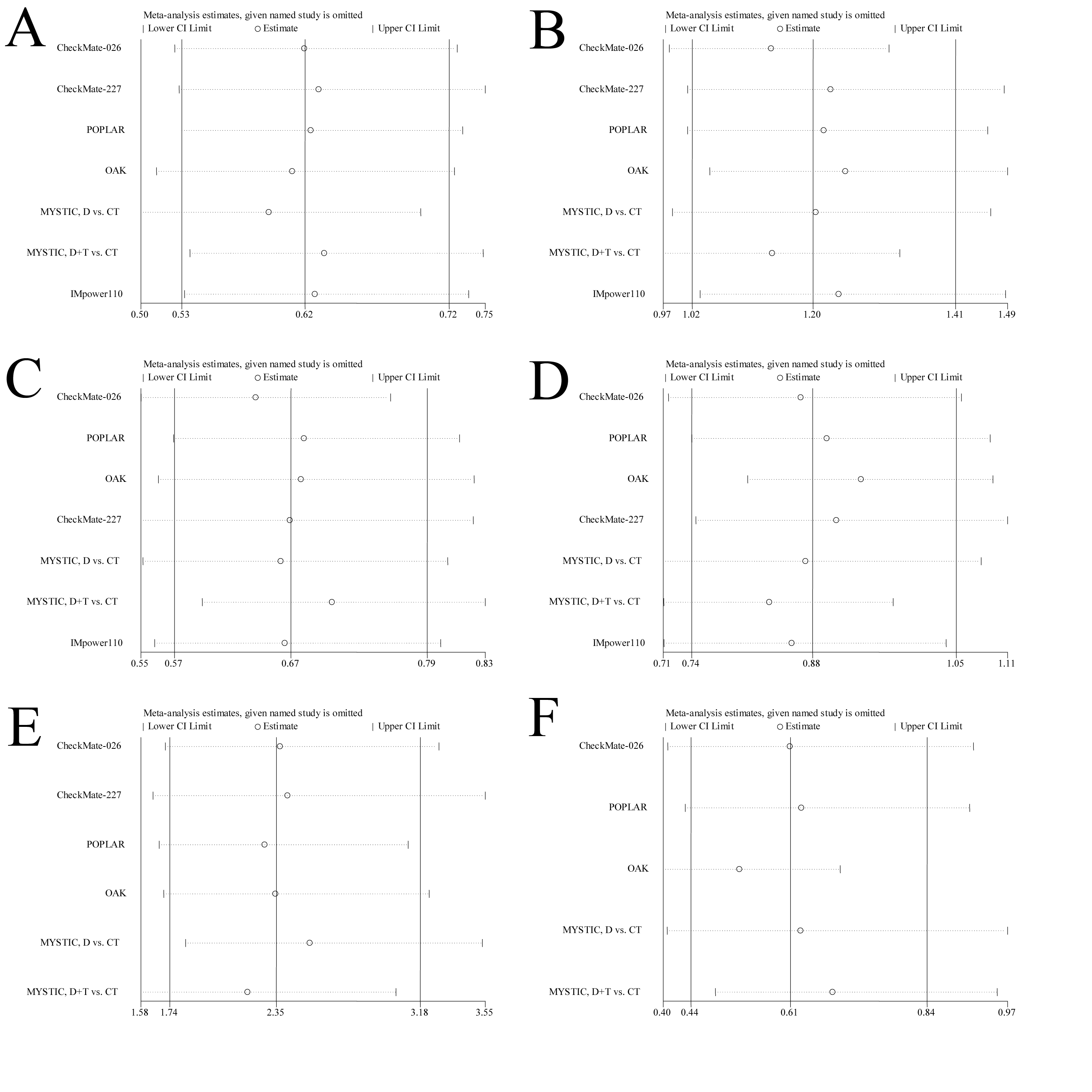

Supplement: S1 Fig — (A) PFS in high-TMB patients; (B) PFS in low-TMB patients; (C) OS in high-TMB patients; (D) OS in low-TMB patients; (E) ORR in high-TMB patients; (F) ORR in low-TMB patients. (TIF) [file pone.0263629.s002.tif]

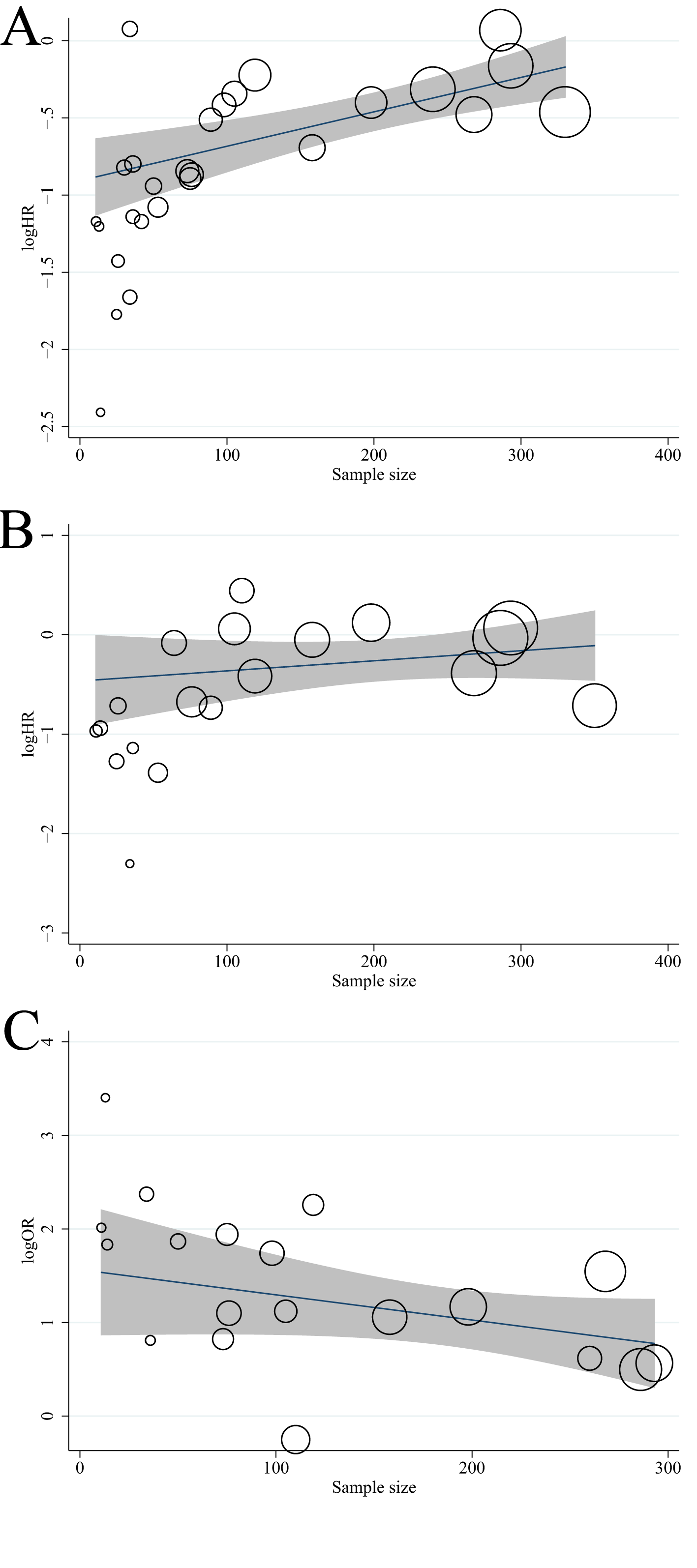

Supplement: S2 Fig — (TIF) [file pone.0263629.s003.tif]

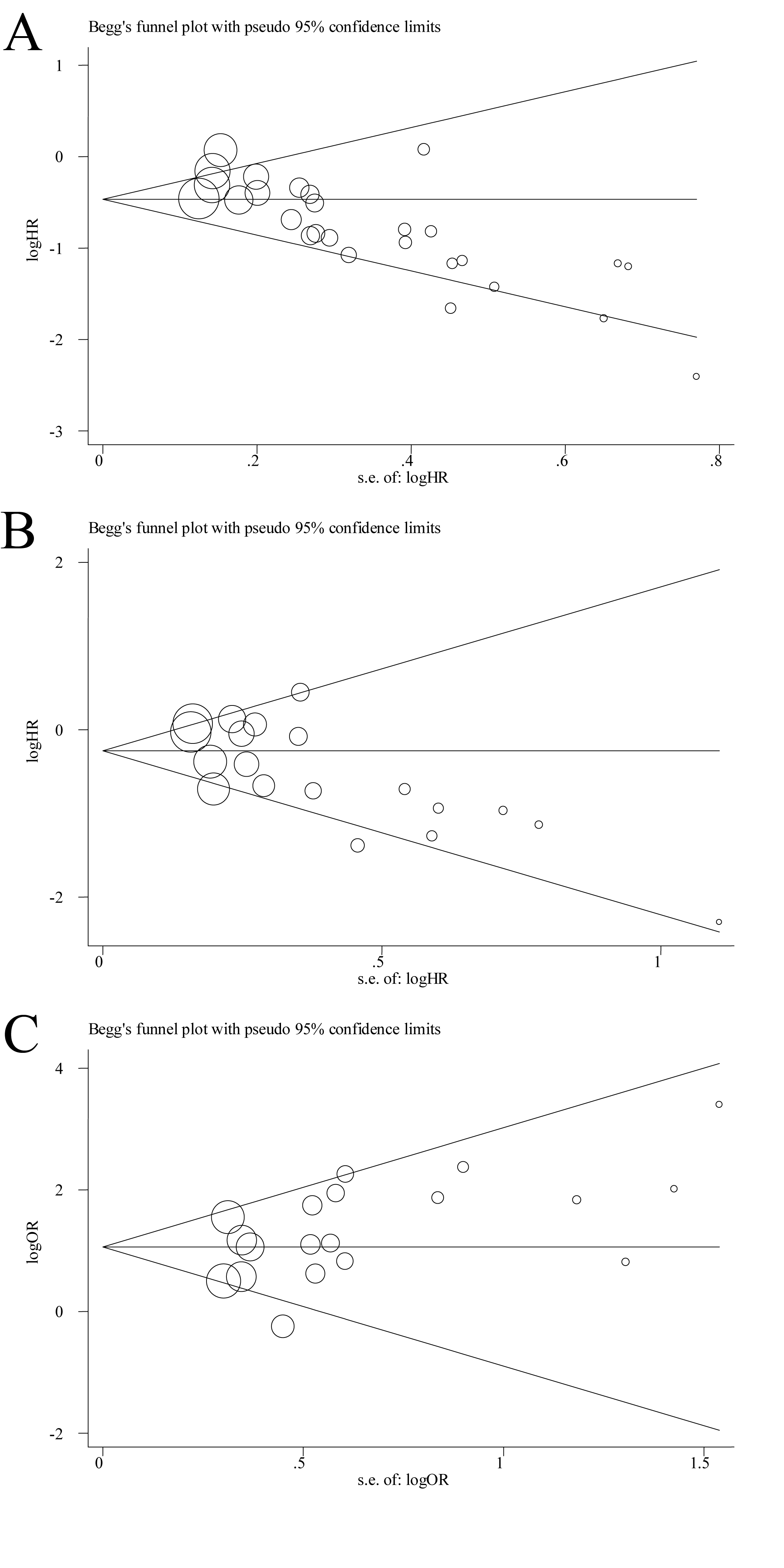

Supplement: S3 Fig — (TIF) [file pone.0263629.s004.tif]

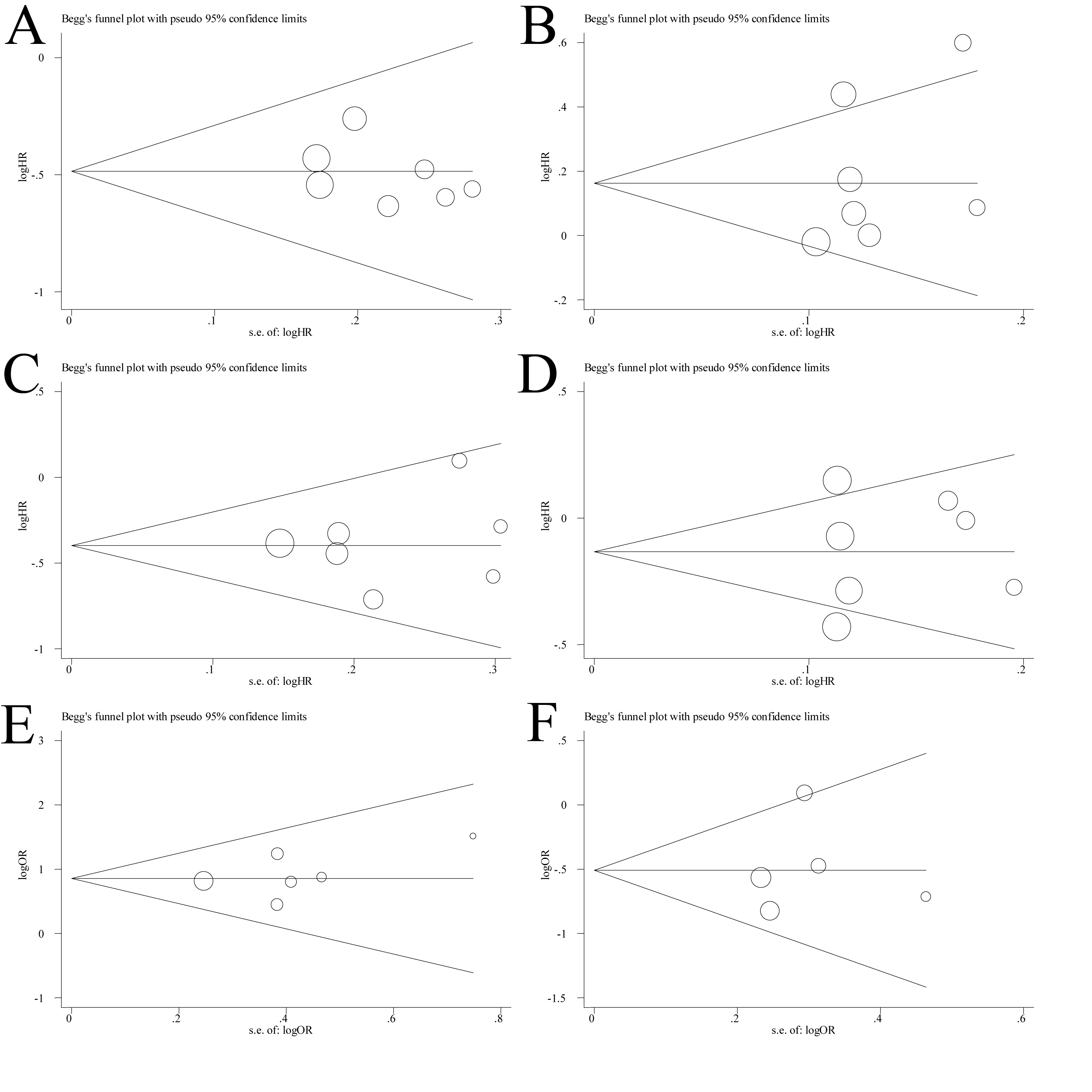

Supplement: S4 Fig — (A) PFS in high-TMB patients; (B) PFS in low-TMB patients; (C) OS in high-TMB patients; (D) OS in low-TMB patients; (E) ORR in high-TMB patients; (F) ORR in low-TMB patients. (TIF) [file pone.0263629.s005.tif]

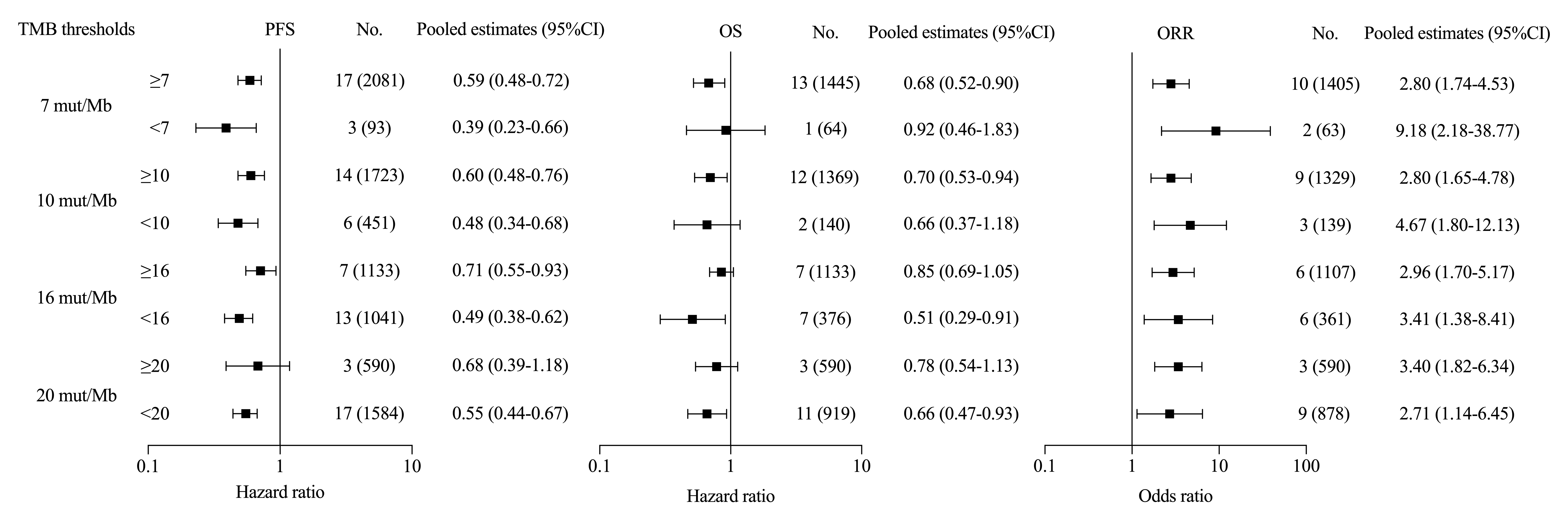

Supplement: S5 Fig — (TIFF) [file pone.0263629.s006.tiff]

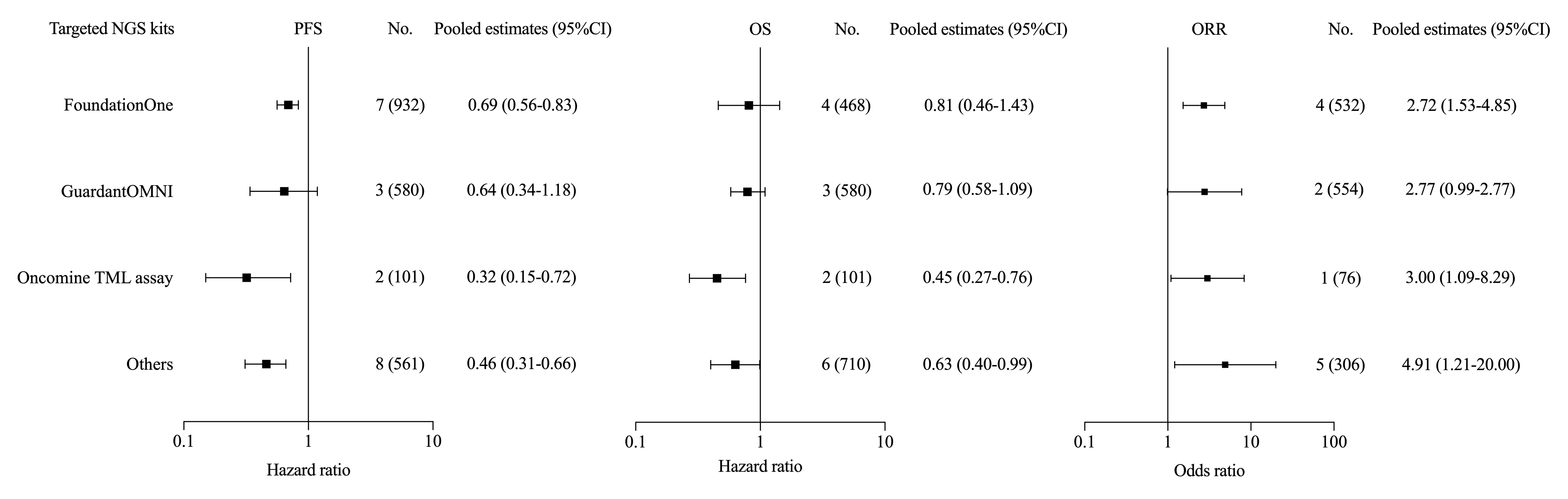

Supplement: S6 Fig — (TIFF) [file pone.0263629.s007.tiff]
