# Supplementary material for: Predictive value of tumor mutational burden for immunotherapy in non-small cell lung cancer: A systematic review and meta-analysis
Source: PLoS One. 2022 Feb 3;17(2):e0263629. doi: 10.1371/journal.pone.0263629 (PMC8812984; doi:10.1371/journal.pone.0263629)
Supplement: S1 Table — (DOCX) [file pone.0263629.s008.docx]

S1 Table. NOS assessment of studies in meta-analysis of high TMB versus low TMB in NSCLC patients treated with immunotherapy

| Study | Selection | | | | Comparability | Outcome | | | Total scores |
| --- | --- | --- | --- | --- | --- | --- | --- | --- | --- |
|  | Representativeness of the exposed cohort | Selection of the non-exposed cohort | Ascertainment of exposure | Demonstration that outcome of interest was not present at start of study | Comparability of cohorts on the basis of the design or analysis | Assessment of outcome | Was follow-up long enough for outcome to occur | Adequacy of follow-up of cohorts |  |
| Rizvi N (2015) | * | * | * | * |  | * | * | * | 7 |
| Carbone D (2017) | * | * | * | * |  | * | * | * | 7 |
| Goodman A (2017) | * | * | * | * |  | * | * | * | 7 |
| Rizvi H (2018) | * | * | * | * |  | * | * | * | 7 |
| Hellmann M, CheckMate-012 (2018) | * | * | * | * | * | * | * | * | 8 |
| Hellmann M, CheckMate-227 (2018) | * | * | * | * |  | * | * | * | 7 |
| Gandara D (2018) | * | * | * | * | * | * | * | * | 8 |
| Chae Y (2019) | * | * | * | * | * | * | * | * | 8 |
| Samstein R (2019) | * | * | * | * |  | * | * | * | 7 |
| Ready N (2019) | * | * | * | * |  | * | * | * | 7 |
| Wang Z (2019) | * | * | * | * | ** | * | * | * | 9 |
| Fang W (2019) | * | * | * | * |  | * | * | * | 7 |
| Ohue Y (2019) | * | * | * | * |  | * | * | * | 7 |
| Heeke S (2019) | * | * | * | * | * | * | * | * | 8 |
| Alborelli I (2020) | * | * | * | * | ** | * | * | * | 9 |
| Wang Z (2020) | * | * | * | * |  | * | * | * | 7 |
| Hurkmans D (2020) | * | * | * | * |  | * | * | * | 7 |
| Huang D (2020) | * | * | * | * | ** | * | * | * | 9 |
| Aggarwal C (2020) | * | * | * | * |  | * | * | * | 7 |
| Rizvi N (2020) | * | * | * | * | ** | * | * | * | 9 |
| Shim J (2020) | * | * | * | * |  | * | * | * | 7 |
| Xu Y (2020) | * | * | * | * | * | * | * | * | 8 |
| B-F1RST study (2020) | * | * | * | * |  | * | * | * | 7 |
| Chen X (2021) | * | * | * | * |  | * | * | * | 7 |
| Ma Y (2021) | * | * | * | * | * | * | * | * | 8 |
| Pabla S (2021) | * | * | * | * |  | * | * | * | 7 |
| Kim H (2021) | * | * | * | * |  | * | * | * | 7 |
| Yoh K (2021) | * | * | * | * |  | * | * | * | 7 |
