# Supplementary material for: Predictive value of tumor mutational burden for immunotherapy in non-small cell lung cancer: A systematic review and meta-analysis
Source: PLoS One. 2022 Feb 3;17(2):e0263629. doi: 10.1371/journal.pone.0263629 (PMC8812984; doi:10.1371/journal.pone.0263629)
Supplement: S2 Table — (DOCX) [file pone.0263629.s009.docx]

S2 Table. Quality assessment of studies in meta-analysis of immunotherapy versus chemotherapy using Cochrane Collaboration’s tool for assessing risk of bias

| Trial | Random sequence generation (selection bias) | Allocation concealment (selection bias) | Blinding of participants and personnel (performance bias) | Blinding of outcome assessment (detection bias) | Incomplete outcome data (attrition bias) | Selective reporting (reporting bias) |
| --- | --- | --- | --- | --- | --- | --- |
| CheckMate-026 | Low | Low | High | Low | Low | Low |
| CheckMate-227 | Low | Low | High | Low | Low | Low |
| POPLAR | Low | High | High | Unclear | Low | Low |
| OAK | Low | High | High | Unclear | Low | Low |
| MYSTIC | Low | Low | High | Low | Low | Low |
| IMpower110 | Low | Low | High | Unclear | Low | Low |
