# Supplementary material for: Predictive value of tumor mutational burden for immunotherapy in non-small cell lung cancer: A systematic review and meta-analysis
Source: PLoS One. 2022 Feb 3;17(2):e0263629. doi: 10.1371/journal.pone.0263629 (PMC8812984; doi:10.1371/journal.pone.0263629)
Supplement: S3 Table — (DOCX) [file pone.0263629.s010.docx]

S3 Table. Survival and response outcomes in meta-analysis of high TMB versus low TMB group in NSCLC patients receiving immunotherapy

| Study | PFS, HR (95%CI) | OS, HR (95%CI) | ORR, n/total (%) | |
| --- | --- | --- | --- | --- |
|  |  |  | High TMB group | Low TMB group |
| Rizvi N (2015) | 0.19 (0.08-0.47) | NA | 10/17 (58.8%) | 2/17 (11.8%) |
| Carbone D (2017) | 0.50 (0.31-0.81)^#^ | 0.95 (0.58-1.54)^#^ | 22/47 (46.8%) | 26/111 (23.4%) |
| Goodman A (2017) | 0.32 (0.13-0.81) | 0.32 (0.07-1.50) | 1/3 (33.3%) | 6/33 (18.2%) |
| Rizvi H (2018) | 0.73 (0.55-0.96) | NA | NA | NA |
| Hellmann M, CheckMate-012 (2018) | 0.41 (0.23-0.73) | NA | 19/37 (51.4%) | 5/38 (13.2%) |
| Hellmann M, CheckMate-227 (2018) | 0.63 (0.49-0.80)^#^ | NA | NA | NA |
| Gandara D, OAK trial (2018) | 0.85 (0.64-1.12)^&^ | 1.07 (0.78-1.47)^&^ | 16/77 (20.8%) | 28/216 (13.0%) |
| Gandara D, POPLAR trial (2018) | 0.71 (0.43-1.17)^&^ | 1.06 (0.62-1.81)^&^ | 7/25 (28.0%) | 9/80 (11.3%) |
| Chae Y (2019) | 1.08 (0.48-2.46) | 0.10 (0.01-0.76) | NA | NA |
| Samstein R (2019) | NA | 0.49 (0.33-0.72)^&^ | NA | NA |
| Ready N (2019) | 0.66 (0.39-1.12)^#^ | NA | 21/48 (43.8%) | 6/50 (12.0%) |
| Wang Z (2019) | 0.39 (0.18-0.84) | NA | 11/28 (39.3%) | 2/22 (9.1%) |
| Chae Y (2019) | 3.50 (1.10-11.2) | 4.8 (1.30-18.1) | NA | NA |
| Fang W (2019) | 0.43 (0.25-0.74) | NA | 7/25 (28.0%) | 7/48 (14.6%) |
| Ohue Y (2019) | 0.31 (0.08-1.10) | 0.38 (0.09-1.50) | 3/4 (75.0%) | 2/7 (28.6%) |
| Heeke S (2019) | 0.45 (0.20-0.93) | NA | NA | NA |
| Alborelli I (2020) | 0.42 (0.25-0.72) | 0.51 (0.29-0.90) | 12/25 (48.0%) | 12/51 (23.5%) |
| Wang Z (2020) | NA | 0.92 (0.46-1.82) | NA | NA |
| Hurkmans D (2020) | 0.17 (0.05-0.64)^#^ | 0.28 (0.09-0.91)^#^ | NA | NA |
| Huang D (2020) | 0.09 (0.02-0.41) | 0.39 (0.12-1.27) | 5/7 (71.4%) | 2/7 (28.6%) |
| Aggarwal C (2020) | 0.24 (0.09-0.66) | 0.49 (0.17-1.42) | NA | NA |
| Rizvi N, D mono (2020) | 1.07 (0.79-1.44)^#^ | 0.97 (0.71-1.32)^#^ | 23/77 (29.9%) | 43/209 (20.6%) |
| Rizvi N, D+T (2020) | 0.62 (0.44-0.88)^#^ | 0.68 (0.46-0.98)^#^ | 31/64 (48.4%) | 34/204 (16.7%) |
| Shim J, cohort 1 (2020) | 0.67 (0.45-0.99) | 1.13 (0.72-1.79) | 24/47 (51.1%) | 24/47 (24.5%) |
| Shim J, cohort 2 (2020) | 0.60 (0.35-1.03) | 0.48 (0.23-1.01) | NA | NA |
| Xu Y (2020) | 0.34 (0.18-0.63) | 0.25 (0.10-0.60) | NA | NA |
| B-F1RST study (2020) | 0.80 (0.54-1.18) | 0.66 (0.40-1.10) | 10/28 (35.7%) | 5/91 (5.5%) |
| Chen X (2021) | 0.31 (0.13-0.77)^#^ | NA | NA | NA |
| Ma Y (2021) | 0.30 (0.08-1.16) | NA | 5/6 (83.3%) | 1/7 (14.3%) |
| Pabla S (2021) | NA | 1.56 (0.78-3.13) | 12/56 (21.4%) | 14/54 (25.9%) |
| Kim H (2021) | 0.44 (0.19-1.01)^#^ | NA | 8/33 (24.2%) | 10/68 (14.7%) |

^#^ HR estimates were calculated using data extracted from survival curves.

^&^ HR estimates were calculated using original data.

NSCLC: non-small cell lung cancer; TMB: tumor mutation burden; PFS: progression-free survival; OS: overall survival; ORR: objective response rate; HR: hazard ratio; 95%CI: 95% confidence interval; NA: not available.
