# Supplementary material for: Predictive value of tumor mutational burden for immunotherapy in non-small cell lung cancer: A systematic review and meta-analysis
Source: PLoS One. 2022 Feb 3;17(2):e0263629. doi: 10.1371/journal.pone.0263629 (PMC8812984; doi:10.1371/journal.pone.0263629)
Supplement: S4 Table — (DOCX) [file pone.0263629.s011.docx]

S4 Table. Survival and response outcomes in meta-analysis of immunotherapy versus chemotherapy in NSCLC patients with high or low TMB.

| Trials | PFS, HR (95%CI) | |  | OS, HR (95%CI) | |  | ORR, n/total (%) | |
| --- | --- | --- | --- | --- | --- | --- | --- | --- |
|  | High TMB | Low TMB |  | High TMB | Low TMB |  | High TMB | Low TMB |
| CheckMate-026 | 0.62 (0.38-1.00) | 1.82 (1.30-2.55) |  | 1.10 (0.64-1.88) | 0.99 (0.71-1.40) |  | 22/47 (46.8%) vs. 17/60 (28.3%) | 26/111 (23.4%) vs. 31/94 (33.0%) |
| CheckMate-227 | 0.58 (0.41-0.81) | 1.07 (0.84-1.35) |  | 0.68 (0.51-0.91) | 0.75 (0.59-0.94) |  | 63/139 (45.3%) vs. 43/160 (26.9%) | NA |
| POPLAR | 0.57 (0.33-0.99) | 1.09 (0.77-1.55) |  | 0.56 (0.31-1.00) | 0.76 (0.52-1.12) |  | 7/25 (28.0%) vs. 3/38 (7.9%) | 9/80 (11.3%) vs. 14/68 (20.6%) |
| OAK | 0.65 (0.47-0.92) | 0.98 (0.80-1.20) |  | 0.64 (0.44-0.92) | 0.65 (0.52-0.81) |  | 16/77 (20.8%) vs. 8/81 (9.9%) | 28/216 (13.0%) vs. 25/209 (12.0%) |
| MYSTIC, D vs. CT^#^ | 0.77 (0.52-1.13) | 1.19 (0.94-1.50) |  | 0.72 (0.50-1.05) | 0.93 (0.74-1.16) |  | 23/77 (29.9%) vs. 15/70 (21.4%) | 43/209 (20.6%) vs. 58/185 (31.4%) |
| MYSTIC, D+T vs. CT^#^ | 0.52 (0.34-0.81) | 1.55 (1.23-1.94) |  | 0.49 (0.32-0.74) | 1.16 (0.93-1.45) |  | 31/64 (48.4%) vs. 15/70 (21.4%) | 34/204 (16.7%) vs. 58/185 (31.4%) |
| IMpower110 | 0.55 (0.33-0.92) | 1.00 (0.78-1.29) |  | 0.75 (0.41-1.35) | 1.07 (0.77-1.47) |  | NA | NA |

# D and D+T indicate durvalumab and durvalumab plus tremelimumab, respectively. CT: chemotherapy; NSCLC: non-small cell lung cancer; TMB: tumor mutation burden; PFS: progression-free survival; OS: overall survival; ORR: objective response rate; HR: hazard ratio; 95%CI: 95% confidence interval; NA: not available.
