# Supplementary material for: Predictive value of tumor mutational burden for immunotherapy in non-small cell lung cancer: A systematic review and meta-analysis
Source: PLoS One. 2022 Feb 3;17(2):e0263629. doi: 10.1371/journal.pone.0263629 (PMC8812984; doi:10.1371/journal.pone.0263629)
Supplement: S5 Table — (DOCX) [file pone.0263629.s012.docx]

S5 Table. Subgroup analysis of immunotherapy versus chemotherapy in NSCLC patients with high or low TMB.

| Subgroup |  | PFS |  |  |  | OS |  |  |  | ORR |  |
| --- | --- | --- | --- | --- | --- | --- | --- | --- | --- | --- | --- |
|  | I^2^ (%) | HR (95%CI) | P |  | I^2^ (%) | HR (95%CI) | P |  | I^2^ (%) | OR (95%CI) | P |
| **High TMB** |  |  |  |  |  |  |  |  |  |  |  |
| ICIs category |  |  |  |  |  |  |  |  |  |  |  |
| PD-1/PD-L1 inhibitors | 0 | 0.65 (0.53-0.78) | <0.001 |  | 0 | 0.72 (0.59-0.88) | 0.002 |  | 0 | 2.13 (1.36-3.34) | 0.001 |
| PD-(L)1 inhibitors plus CTLA-4 inhibitors | 0 | 0.56 (0.43-0.73) | <0.001 |  | 37.1 | 0.60 (0.44-0.82) | 0.001 |  | 0 | 2.55 (1.70-3.83) | <0.001 |
| Therapy line |  |  |  |  |  |  |  |  |  |  |  |
| 1 | 0 | 0.61 (0.51-0.74) | <0.001 |  | 28.3 | 0.70 (0.56-0.87) | 0.001 |  | 0 | 2.27 (1.64-3.15) | <0.001 |
| >1 | 0 | 0.63 (0.47-0.84) | 0.001 |  | 0 | 0.62 (0.45-0.84) | 0.002 |  | 0 | 2.86 (1.32-6.22) | 0.008 |
| Sample source |  |  |  |  |  |  |  |  |  |  |  |
| tumor | 0 | 0.59 (0.45-0.78) | <0.001 |  | 57.9 | 0.82 (0.52-1.29) | 0.390 |  | 0 | 2.25 (1.49-3.40) | <0.001 |
| Blood | 0 | 0.63 (0.52-0.76) | <0.001 |  | 0 | 0.63 (0.51-0.76) | <0.001 |  | 0 | 2.48 (1.60-3.84) | <0.001 |
| TMB detected by targeted NGS | 0 | 0.62 (0.52-0.73) | <0.001 |  | 0 | 0.64 (0.55-0.76) | <0.001 |  | 0 | 2.38 (1.72-3.29) | <0.001 |
| **Low TMB** |  |  |  |  |  |  |  |  |  |  |  |
| ICIs category |  |  |  |  |  |  |  |  |  |  |  |
| PD-1/PD-L1 inhibitors | 62.6 | 1.16 (0.95-1.41) | 0.140 |  | 56.7 | 0.86 (0.70-1.04) | 0.120 |  | 21.6 | 0.68 (0.48-0.95) | 0.025 |
| PD-(L)1 inhibitors plus CTLA-4 inhibitors | 79.5 | 1.29 (0.90-1.85) | 0.170 |  | 85.8 | 0.93 (0.61-1.43) | 0.755 |  | - | 0.44 (0.27-0.71) | 0.001 |
| Therapy line |  |  |  |  |  |  |  |  |  |  |  |
| 1 | 69.3 | 1.27 (1.04-1.56) | 0.020 |  | 47.7 | 0.96 (0.82-1.13) | 0.647 |  | 0 | 0.53 (0.39-0.71) | <0.001 |
| >1 | 0 | 1.01 (0.84-1.20) | 0.943 |  | 0 | 0.68 (0.56-0.82) | <0.001 |  | 53.7 | 0.79 (0.37-1.72) | 0.557 |
| Sample source |  |  |  |  |  |  |  |  |  |  |  |
| Tumor | 84.3 | 1.38 (0.82-2.31) | 0.229 |  | 42.8 | 0.84 (0.64-1.09) | 0.191 |  | - | 0.62 (0.4-1.15) | 0.130 |
| Blood | 61.3 | 1.15 (0.96-1.37) | 0.120 |  | 73.7 | 0.90 (0.71-1.13) | 0.351 |  | 50.8 | 0.61(0.40-0.91) | 0.016 |
| TMB detected by targeted NGS | 52.9 | 1.13 (0.98-1.31) | 0.089 |  | 70.7 | 0.87 (0.72-1.05) | 0.153 |  | 50.8 | 0.61 (0.40-0.91) | 0.016 |

NSCLC: non-small cell lung cancer; ICIs: immune checkpoint inhibitors; PD-1: programmed cell death 1; PD-L1: programmed cell death-ligand 1; CTLA-4: cytotoxic T lymphocyte associated antigen 4; TMB: tumor mutation burden; NGS: next-generation sequencing; PFS: progression-free survival; OS: overall survival; ORR: objective response rate; mut/Mb: numbers of mutation per Megabase; HR: hazard ratio; OR: odds ratio; 95%CI: 95% confidence interval.
